# Assessing the Effects of Acupuncture by Comparing Needling the Hegu Acupoint and Needling Nearby Nonacupoints by Spectral Analysis of Microcirculatory Laser Doppler Signals

**DOI:** 10.1093/ecam/neq073

**Published:** 2011-06-16

**Authors:** Hsin Hsiu, Wei-Chen Hsu, Chia-Liang Hsu, Shih-Min Huang

**Affiliations:** ^1^Graduate Institute of Biomedical Engineering, National Taiwan University of Science and Technology, Taipei 10607, Taiwan; ^2^Department of Chinese Medicine, Taipei City Hospital RenAi Branch, Taipei, Taiwan; ^3^Department of Electrical Engineering, Yuan Ze University, Taoyuan, Taiwan

## Abstract

We aimed to assess the effects of acupuncture by analyzing the frequency content of skin blood-flow signals simultaneously recorded at the Hegu acupoint and two nearby nonacupoints following acupuncture stimulation (AS). Laser Doppler flowmetry (LDF) signals were measured in male healthy volunteers in two groups of experiments: needling the Hegu acupoint (*n* = 13) and needling a nearby nonacupoint (control experiment; *n* = 10). Each experiment involved recording a 20 min baseline-data sequence and two sets of effects data recorded 0–20 and 50–70 min after stopping AS. Wavelet transform with Morlet mother wavelet was applied to the measured LDF signals. Needling the Hegu acupoint significantly increased the blood flow, significantly decreased the relative energy contribution at 0.02–0.06 Hz and significantly increased the relative energy contribution at 0.4–1.6 Hz at Hegu, but induced no significant changes at the nonacupoints. Also, needling a nearby nonacupoint had no effect in any band at any site. This is the first time that spectral analysis has been used to investigate the microcirculatory blood-flow responses induced by AS, and has revealed possible differences in sympathetic nerve activities between needling the Hegu acupoint and its nearby nonacupoint. One possible weakness of the present design is that different De-Qi feelings following AS could lead to nonblind experimental setup, which may bias the comparison between needling Hegu and its nearby nonacupoint. Our results suggest that the described noninvasive method can be used to evaluate sympathetic control of peripheral vascular activity, which might be useful for studying the therapeutic effects of AS.

## 1. Introduction

Acupuncture therapy, which forms an essential part of oriental medicine, has been suggested to prevent or treat illness by adjusting autonomic functions via the autonomic nervous system, hormonal system or neuroendocrine system [1, 2]. It has also been found that the microcirculatory perfusion of the skin surface [2–7] and the internal organs [2, 8–10] can be changed after acupuncture stimulation (AS).

Laser Doppler flowmetry (LDF) is increasingly being used for monitoring the microcirculation due to its advantages of a good frequency response, ease of application, continuity and tissue specificity, and is therefore well suited for noninvasive investigations of microvascular responses to acupuncture [3, 8, 11]. Spectral analysis of LDF signals reveals that blood-flow oscillations at frequencies from 0.01 to 1.6 Hz might reflect various physiological rhythms. Periodic oscillations with characteristic frequency peaks observed within the following frequency bands, 0.0095–0.02, 0.02–0.06, 0.06–0.15, 0.15–0.4 and 0.4–1.6 Hz, are suggested to be influenced by the endothelial activity of the vessel wall, the neurogenic activity of the vessel wall, the intrinsic myogenic activity of vascular smooth muscle, the respiration and the heartbeat, respectively [12, 13]. Calculating the occupied proportions of spectral power for each frequency bands may help to monitor the activities of different physiological rhythms, and hence provides a promising tool to study the underlying microcirculatory regulatory mechanism [14, 15].

We have previously shown that the microcirculatory characteristics as monitored by LDF differ between acupoints and nonacupoints [15, 16]. It has been noted that AS can increase the microcirculatory blood flow at the acupoints [1, 3, 5]. The aim of the present study was to assess the effects of acupuncture by analyzing the frequency content of skin blood-flow signals simultaneously recorded at the Hegu acupoint (which is an important acupoint in oriental medicine) and two nearby nonacupoints following AS. Spectral analysis can reveal the characteristic frequency of blood-flow oscillations at the acupoints, and might therefore help in understanding the mechanisms underlying responses to AS.

## 2. Methods

### 2.1. Experimental Setup and Data Acquisition

Experiments were performed on male healthy volunteers aged 20–27 years and without signs or symptoms of cardiovascular or neurological disease. The subjects were all Taiwan natives, were lightly clothed, supine and were allowed to stabilize for at least 20 min before commencing recording. The environmental temperature was within 23–25°C during the entire measuring period. The institutional ethics committee at Taipei City Hospital approved the study protocol, and each volunteer gave informed consent before entering the study. Tea, coffee, alcohol and smoking were forbidden on the day before experiments. All subjects did not exercise or consume food for at least 1 h before each experiment [14, 16, 17].

The electrocardiogram (ECG) and LDF signals were measured simultaneously and noninvasively. ECG signals were measured by surface electrodes, and acquired by a preamplifier (lead II, RA-LL; 6600-series, Gould, USA). LDF (VP1 probe; MBF3, Moor Instruments, UK) was used to measure the microcirculatory flux with a time constant of 0.001 s, a cut-off frequency of 14.9 kHz, and a sampling frequency of 40 Hz at measurement sites between the thumb and the index finger on the back of the left hand. The laser operating wavelength and output power were 780 nm and < 1.6 mW, respectively. Subjects were asked to relax and breathe naturally throughout the measurement period so as to avoid motion artifacts. The signals were connected to an analog-to-digital converter card (PCI-9111DG, Adlink Technology, Taiwan) operating at a sampling rate of 1024 Hz [17–20].

**Figure 1:**
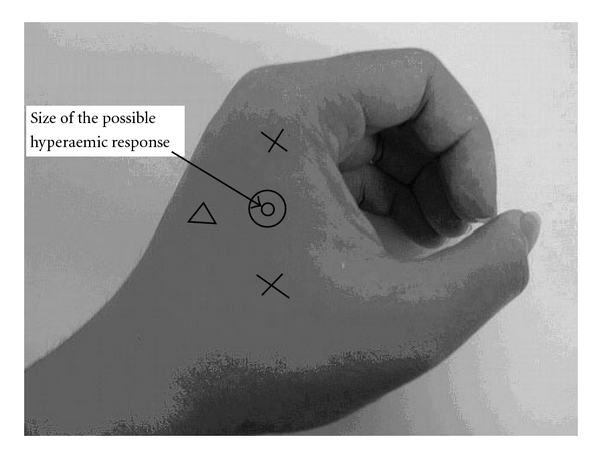
Locations of measurement sites. Measurement sites: “O", Hegu acupoint (Site 1); “X", nearby nonacupoints (lower: Site 2, halfway between Hegu and Yangxi; upper: Site 3, halfway between Hegu and Sanjian); and “open triangle", location of the thermistor. Typical size of the hyperemic response (when it occurred) induced by the inserted needle is indicated. The diameter of the hyperemic response was never larger than 3 mm, which is smaller than the area of the LDF probe head (diameter *∼*6 mm).

The measuring sites of the LDF probe were at left Hegu (LI4, Site 1) and two nearby nonacupoints for comparison: one (Site 2) halfway between Hegu and Yangxi (LI5), and the other (Site 3) halfway between Hegu and Sanjian (LI3). The following two groups of experiments were performed to elucidate the microcirculatory response following AS: (i) Hegu experiment: needling the left Hegu acupoint (Site 1; *n* =13); and (ii) control experiment: needling a nearby nonacupoint (Site 2; *n* =10). The experimental procedures and measurement sites were identical for both experiments except for the location where AS was applied. The LDF probe was held vertically onto the skin surface by a holder with a radius of *∼*6 mm. The chosen measurement sites were located at the back of the hand (Figure 1). Choosing measurement sites away from glabrous skin such as the fingertip [18] or the palm [19] may help to minimize interference from thermoregulatory effects of the arteriovenous shunt vessels. 

During the measurement, the subject was supine on a measurement couch. Each assessment involved making the following acupoint microcirculation (AM) recordings: the 20-min baseline AM (baseline period) was recorded prior to AS, after which the acupuncture needle (stainless steel needle, gauge #30, 7.5 cm in length; Chianhuei, Taiwan) was inserted into the left Hegu acupoint (for the Hegu experiment) or Site 2 (for the control experiment) to a depth of *∼*4 mm for 20 min. The De-Qi feeling following AS was experienced in all the subjects in the Hegu experiment. The needle was then withdrawn to stop AS, with the AM being recorded from 0 to 20 min (early effect) and from 50 to 70 min (later effect) after stopping AS.

Before the baseline and after the later-effect period, we measured fundamental physiological parameters of the subject, including the heart rate (HR), systolic blood pressure (SBP), and diastolic blood pressure (DBP) using a sphygmomanometer (MediGuard 150i, Rossmax, Taiwan). A thermistor was attached to the skin surface to monitor the skin surface temperature near the LDF measurement site (see Figure 1). The resistance of the thermistor was transformed into voltage using a custom-made circuit, which was also sampled by the analog-to-digital converter card. The baseline temperature was 31.8 ± 1.1°C (mean ± SD), and the temperature stability during the baseline period was considered acceptable when the variation was <1.0°C [14].

### 2.2. Signal Analysis

Data files containing spikes that were too large relative to the mean flux value were discarded, since this implied the presence of motion artifacts. The mean, standard deviation (SD) and coefficient of variance (CV, SD/mean) of the HR were calculated in each sequence. In order to ensure that subjects were physiologically stable (so as to avoid interference from microcirculatory autoregulation), the chosen baseline-data sequences had to satisfy the following criteria: (i) CV of the HR <8%, (ii) DBP >60 mmHg, (iii) SBP <130 mmHg and (iv) variations in LDF flux signals <25% [17].

For the LDF signal, the mean microcirculatory blood flow (MBF)—which was defined as the average value of the LDF signals during each 20-min measurement period—was calculated to elucidate the effect of AS on the microcirculation.

Oscillations of the microvascular blood perfusion signal can be separated into different components by spectral analysis [12, 13, 21]. Wavelet transform with Morlet mother wavelet was applied to the measured LDF signals to improve the low-frequency resolution. Periodic oscillations with five characteristic frequency peaks were observed from 0.0095 to 1.6 Hz, with the positions of these peaks falling within the following frequency bands: 0.0095–0.02, 0.02–0.06, 0.06–0.15, 0.15–0.4 and 0.4–1.6 Hz (defined as FR1–FR5, resp.); which are suggested to be influenced by the endothelial activity of the vessel wall, the neurogenic activity of the vessel wall, the intrinsic myogenic activity of vascular smooth muscle, the respiration and the heartbeat, respectively [12, 13]. The energy density within each frequency band was calculated, and the relative energy contribution (REC) in each frequency band (from FR1 to FR5) was defined as the ratio between the total energy density within that band and the total energy density of the entire spectrum from 0.0095 to 1.6 Hz [21].

All signal processing was performed with MATLAB (MathWorks, Natick, MA, USA). Two-tailed paired *t*-test or simple regression analysis were used to verify the statistical significance. Differences were considered significant when *P* < .05.

## 3. Results

### 3.1. Fundamental Physiological Parameters

In Hegu experiment, HR, SBP and DBP were 75 ± 10 beats per minute, 111.2 ± 6.7 and 72.6 ± 5.9 mmHg before the LDF measurement, and were 71 ± 10 beats per minute, 111.5 ± 7.2 and 72.1 ± 4.7 mmHg, respectively, after the LDF measurement. In control experiment, HR, SBP and DBP were 73 ± 8 beats per minute, 111.5 ± 6.3 and 70.8 ± 5.4 mmHg before the LDF measurement, and were 72 ± 9 beats per minute, 112.0 ± 7.2 and 71.7 ± 5.9 mmHg, respectively, after the LDF measurement. These basic physiological parameters did not change significantly between before and after the LDF measurement, except for a significant decrease in the HR in the Hegu experiment after the AS (*P* < .05 by two-tailed paired *t*-test).

### 3.2. MBF Response

**Figure 2:**
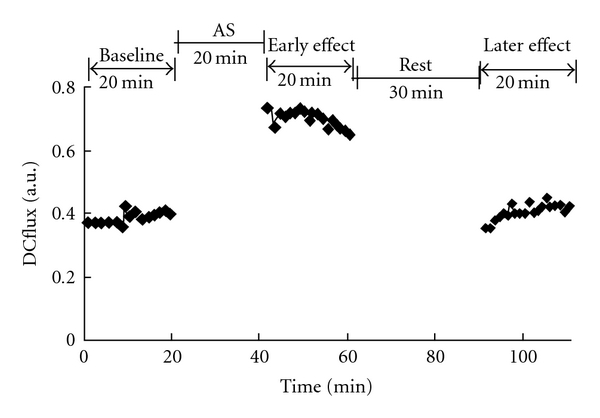
Experimental setup. For the LDF signal, each data point represents the value averaged over a 1-min epoch. At the Hegu acupoint, the MBF increased prominently during the early effect, and then tended to return to the baseline value during the later effect.

**Figure 3:**
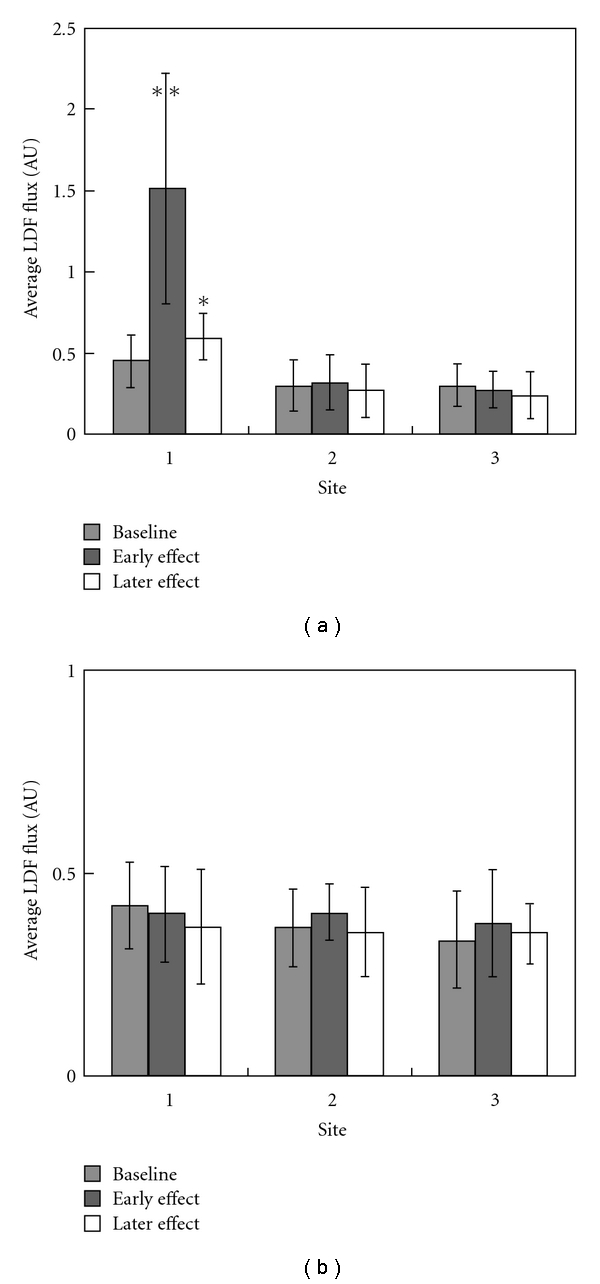
Comparison of the MBF between acupoints and nonacupoints. (a) Hegu experiment (*n* =13) and (b) control experiment (*n* =10). MBF is defined as the average value of LDF flux signal. Data are mean and SD values. *P < .05 and **P < .01, compared with the baseline value by two-tailed paired *t*-test. In the Hegu experiment, both effects at Hegu were significantly larger than the baseline value, whereas there were no significant effects at the nonacupoints. In contrast to the early effect, the later effect at Hegu tended to return to the baseline value. In the control experiment, there were no significant changes in the MBFs at the three sites.

A typical experimental setup is shown in Figure 2. The MBF is compared between acupoints and nonacupoints in Figure 3. Compared to the baseline values, the MBF was significantly increased after the AS (in both the early and later effects) only at Hegu (both *P* < .01 by two-tailed paired *t*-test) in the Hegu experiment. In the control experiments, there were no significant changes for the MBF at all three sites (*P* > .2 by two-tailed paired *t*-test). 

### 3.3. LDF Flux Spectra

**Figure 4:**
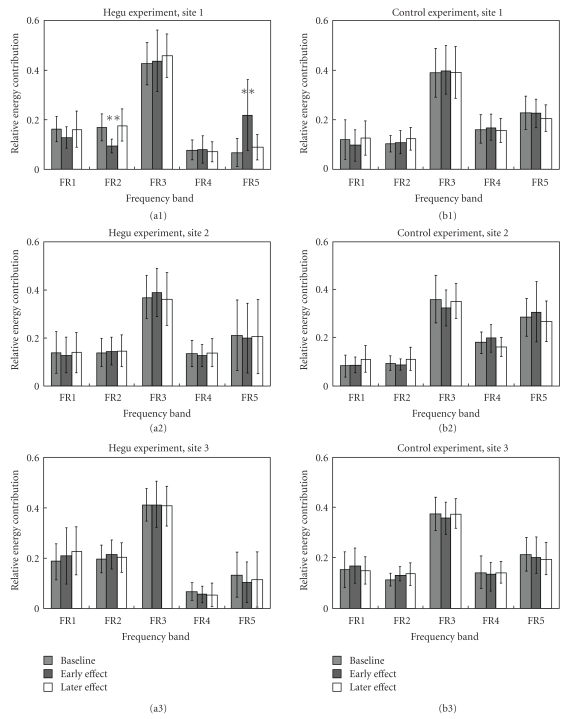
Changes in RECs in the LDF spectra at the three measurement sites. Stimulation and measurement sites: (a1) needling Hegu (*n* = 13), Site 1; (a2) needling Hegu, Site 2; (a3) needling Hegu, Site 3; (b1) control (*n* = 10), Site 1; (b2) control, Site 2; and (b3) control, Site 3. The REC of each frequency band was defined as the total energy density within a particular frequency band divided by the total energy density of the entire spectrum from 0.0095 to 1.6 Hz. *P < .05 and **P < .01, compared with the baseline value by two-tailed paired *t*-test. At Hegu in the Hegu experiment, the REC was significantly decreased at FR2 and was significantly increased at FR5 during the early effect; RECs then moved towards their baseline values during the later effect. There were no significant changes in any band at the nonacupoints in the Hegu experiment and at all three sites in the control experiment.

Changes in the spectra of blood-flow signals following AS at the three measurement sites are compared in Figure 4 In the Hegu experiment, the REC of FR2 was significantly decreased and that of FR5 was significantly increased during the early effect. The powers in these two bands tended to return to their baseline values during the later effect. There were no significant changes in any band after the AS at the two nonacupoints in the Hegu experiment and at all three sites in the control experiment. 

**Figure 5:**
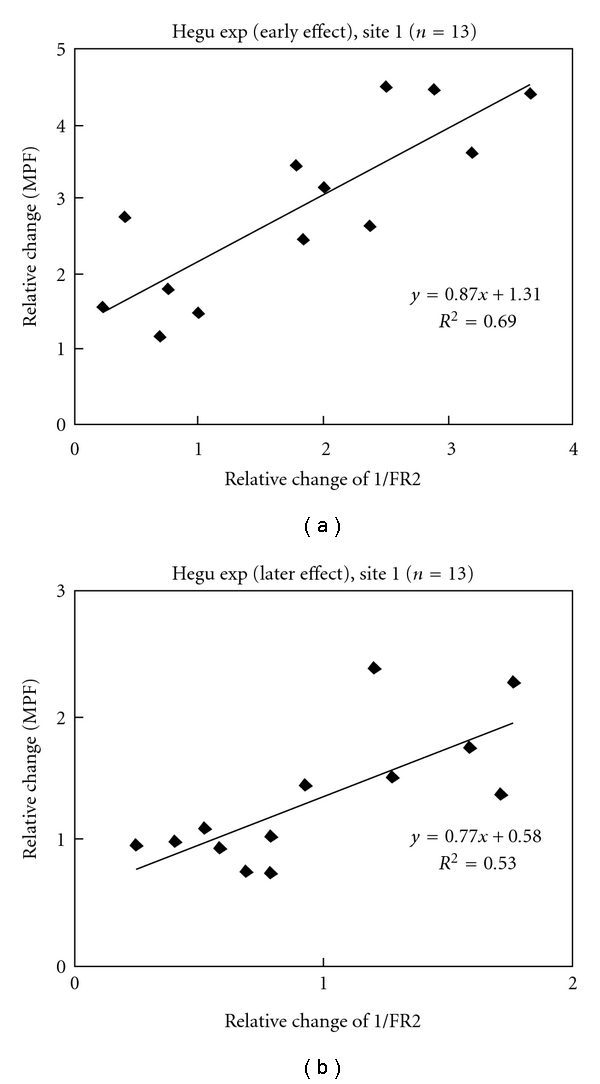
Relation between changes in the MBF and 1/(RECs of FR2) of the LDF signal at Site 1 (Hegu) in the Hegu experiment (*n* =13). (a) Early effect and (b) later effect. Relative changes in the MBF and 1/FR2 of the early and later effects are defined as the value of the early or later effect divided by the average baseline value. In the Hegu experiment, *R* ^2^ were 0.69 and 0.53 (*P* < .01 by *F*-test) during early effect and later effect, respectively, which indicated that the MBF was inversely proportional to the REC of FR2. The *R* ^2^ values were all smaller than 0.05 at the nonacupoints in the Hegu experiment and at all three sites in the control experiment (all *P* > .2 by *F*-test).

As shown in Figure 5, linear regression analysis was performed on the correlation between changes in the MBF and the reciprocal of the REC of FR2. At Hegu in the Hegu experiment, the regression lines had coefficients of determination (*R* ^2^) of 0.69 and 0.53 (*P* < .01 by *F*-test) during early effect and later effect, respectively. It indicated that the MBF was inversely proportional to the REC of FR2. However, the *R* ^2^ values were all smaller than 0.05 at the nonacupoints in the Hegu experiment and at all three sites in the control experiment (all *P* > .2 by *F*-test). 

## 4. Discussion

In this study we analyzed the effects of AS on skin blood flow. The MBF response at Hegu was similar to findings of several previous studies [1, 3, 5]. At Hegu, the blood flow tended to return to the baseline value during the later effect. There were no significant changes in the MBF at the nonacupoints in the Hegu experiment and at all three sites in the control experiment.

**Figure 6:**
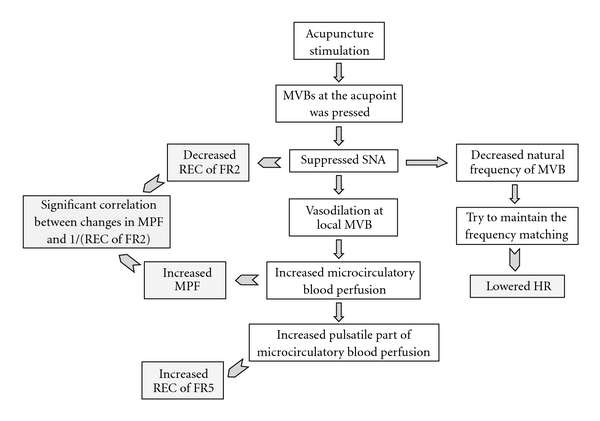
Possible mechanism underlying the changes in the HR and the microcirculatory parameters at the needled site. Applying AS in the Hegu experiment led to significant changes in the HR, the RECs of FR2, and the REC of FR5 at the needled site.

A possible mechanism underlying the changes in the HR and the microcirculatory parameters at the needled site is summarized in Figure 6. The participation of neural mechanisms in the vasodilative effects of AS has been widely studied [2]. The spectral analysis of the blood-flow signal in Figure 4 revealed that in the Hegu experiment, the REC of FR2 at Hegu was significantly decreased after AS, while there were no significant changes at the nearby nonacupoints. The Hegu point is located at one of the thenar muscles and is innervated by sensory and autonomic (mainly sympathetic) fibers [22]. It has been suggested that the oscillations recorded in the blood flow reflect vasomotion, and the sympathetic nervous system plays an important role in regulating vascular tone in the systemic circulation and in isolated vascular beds [23, 24]. FR2 is believed to be the most important frequency range for sympathetic modulation of vasomotor activity at the local microvascular beds (MVBs) in humans [12, 13, 23, 24]. Therefore, the results of this study suggest that one of the important mechanisms underlying the blood-flow response at Hegu is vessel dilation induced by decreased sympathetic nerve activity (SNA). In contrast to the effect at Hegu, there were no significant changes in the REC of FR2 at the two nonacupoints, which might indicate the absence of significant blood-flow changes at these sites. Several previous studies found that AS decreased SNA [25–31]. To the best of our knowledge, no previous study has employed spectral analysis of the LDF signal of skin blood flow to explore the mechanisms involved in the vasodilatory activity induced by AS. The difference in SNA between the Hegu acupoint and nonacupoints suggests that the vascular beds dilate more at Hegu than at the nonacupoints, which helps to concentrate the blood flow into the acupoint. 

Skin temperature [5], thermography [28], microneurography [29], plethysmography [30], HR variability [18] and BP changes [31] have previously been used to evaluate SNA following acupuncture. In the present study, the advantages of the noninvasiveness and real-time capability of LDF measurements facilitated the assessment of SNA differences between an acupoint and its surroundings, which minimized interference effects on the skin blood flow.

Linear regression analysis of the correlation between MBF and 1/(REC of FR2) further strongly support the association between the flux and the SNA responses. Figure 5 illustrates that the decreased SNA, which might dilate the local vessel, is an important factor contributing to the increase in the microcirculatory flux.

In the Hegu experiment, the blood-flow responses differed between Hegu and the nearby nonacupoints, with (i) the changes in the MBF being larger at Hegu, (ii) there being no significant changes in the REC of FR2 at the nonacupoints and (iii) the inverse proportionality between the MBF and the REC of FR2 only being evident at Hegu. It has been noted that acupoints have higher concentrations of neural and vascular elements [32]. The larger changes in the REC of FR2 indicate larger changes in SNA, and imply that the regulation of microcirculatory perfusion of the vascular beds is stronger at the acupoints than at other sites.

The significantly decrease in the HR following AS observed in the Hegu experiment in this study might be another whole-body effect induced by the decreased SNA. A similar effect has been noted in previous studies, which has resulted in acupuncture being used to treat tachycardia [33].

In the Hegu experiment, the REC of FR5 significantly increased at Hegu but not at the nearby nonacupoints. It has been suggested that the REC of FR5 represents the influence of the HR on the blood flow [12, 13], and hence its increase would indicate an increased transmission efficiency for the pulsatile component of the blood flow. Wang Lin et al. [34] suggested that the radial movement of each segment of the arterial wall has a natural frequency. An HR near to the arterial natural frequency will result in the largest area oscillation in the artery, producing the largest output for the pressure pulse and thereby improving the arterial transmission efficiency. This represents a frequency-matching condition between the HR and the arterial system [34]. Decreased SNA is known to lower the HR and dilate the terminal vessel. The decreases in the REC of FR2 and SNA following AS will dilate the terminal vessel, decrease the natural frequency of the vascular bed, and lower the HR so as to maintain the frequency-matching condition [35]. The improving perfusion induced by AS—which is also supported by the increased MBF following AS—might partly account for the therapeutic effects of AS.

It was revealed in Figure 4 that there were no significant changes for the REC of FR1 at the needled site in both experiments. Endothelial cells control the contraction and relaxation of smooth muscle cells by releasing vasodilators (such as nitric oxide and prostacyclin) as well as vasoconstrictors (such as endothelium and platelet-activating factors) [36]. Some vasodilators, such as calcitonin gene-related peptide, substance P and nitric oxide, are suggested to contribute to the vasodilation effects following AS [1, 2, 37]. However, although a vasodilation response was revealed in the Hegu experiment, no confirmed conclusion about the detailed endothelial secretion in the AS effect can be drawn from the present result. It can be our future work to identify which vasoactive substances participate in the microcirculatory response following AS.

In the results of both experiments, there were no significant changes in the RECs of FR3 and FR4. FR3 is believed to correspond to the most important frequency range in myogenic responses at local MVBs in humans [12, 13]. The myogenic response is due to the smooth muscle cells in vessel walls responding continually to changes in intravascular pressure [13]. In both experiments, there were no significant changes in SBP and DBP between before and after the whole measurement procedure. It is possible that following AS, the intravascular pressure did not change prominently, and therefore no significant changes of REC of FR3 occurred in both experiments.

REC of FR4 is suggested to correspond to the influence of the respiratory function. However, since the respiratory function mainly induces whole-body effect, its activity is weakly present in the microvascular blood flow signal [13]. It could be one important reason accounting for the non-significant changes in REC of FR4 following AS in both experiments.

One limitation of the present study is that blood-flow responses could be induced by microtrauma caused by the introduction of a needle into the skin; we attempted to avoid this trauma effect by observing whether the skin surface around the needled site turned red after withdrawing the needle. In the experimental design, the control experiment (in which the needle was inserted into a nearby nonacupoint) was performed in order to clarify the specific effects of AS at Hegu. In contrast to the effects in the Hegu experiment, in the control experiment there were no significant changes in any of the spectral bands (shown in Figure 4) and no significant regression correlation between the relative change in the MBF and the REC of FR2 (shown in Figure 5) at all three sites. Moreover, the effects on the HR differed between the two experiments, with a significant decrease for the Hegu experiment and nonsignificant effect for the control experiment. These results illustrate different microcirculatory responses between needling Hegu and needling a nearby nonacupoint, and imply that changes in the HR and LDF spectrum at Hegu in the Hegu experiment may reflect a substantial change in the underlying physiology.

AS is typically associated with the De-Qi sensation (a special feeling, including distension of soreness or numbness, as described mutually by the subjects and the acupuncturist). The De-Qi sensation has been noted to be associated with enhanced blood flow at the needling site [5], and has been suggested to be important for acupuncture to achieve its effects [38]. In the present study, the De-Qi feeling following AS was experienced in all trials in the Hegu experiment. However, in the control experiment, there were only two subjects (2/10) experiencing the De-Qi-like feeling when needling the nearby nonacupoint. Moreover, the De-Qi-like feelings in the control experiment were less prominent than those experienced in the Hegu experiment. The different feeling following AS could lead to non-blind experimental setup, bias the comparison between the results of Hegu and control experiments, and hence form another weakness of the present experimental design.

A relationship between blood-flow increases and intensity of the AS has noted that a more intense AS can result in a more pronounced blood-flow increases [1, 38]. The De-Qi feeling can be an important indication of the intensive needle stimulation. During De-Qi, the resultant blood-flow increases were suggested to relate to the release of vasodilatatory neuropeptides from nerve endings, and possibly to interactions with sympathetic vasoconstriction neurons [5, 38]. In the present study, the different condition of experiencing De-Qi feeling between the two groups might help to explain the different microcirculatory response when needling Hegu and its nearby nonacupoint. In Hegu experiment, all the subjects experienced the De-Qi sensation, which may induce the more severe suppression of SNA. It could therefore lead to local vasodilation, and hence prominently improve the perfusion condition of the MBF. In contrast, De-Qi feelings were hardly experienced when needling the nearby nonacupoint. It is possible that the activities of the above-mentioned mechanisms (including the suppressed SNA and increased neuropeptides release) were weaker when needling the nearby nonacupoint, and therefore the responses in the MBF and LDF spectrum were less prominent than in the Hegu experiment.

The application of AS can change the microcirculatory perfusion at the needled as well as other acupoints [5]. The MBF plays important roles in circulatory transportation, such as supplying nutrients and removing harmful molecules, and therefore the therapeutic effects by applying AS might be relevant with the improvement extent for the MBF perfusion. Figure 3 revealed that needling Hegu brought a larger improvement for the MBF perfusion, which could partly account for the reason why the acupuncture should be performed on the acupoints rather than arbitrary sites on the skin surface. Moreover, Figure 4 revealed that the decrease in the REC of FR2 was more prominent by needling Hegu than by needling the nearby nonacupoint. Since this result implies inhibition of SNA following AS, the vessel can be more dilated, and hence the perfusion resistance of the MBF can be more improved at the local MVB by needling Hegu than by needling the nearby nonacupoint. Monitoring the blood-flow response or spectrum can therefore aid the evaluation of the therapeutic effects of acupuncture.

A decrease in SNA might lead to vasodilation of the MVB, and hence decrease the elastic modulus of vessel walls. Many previous studies found that changes in the elastic properties of the MVBs influenced arterial wave transmission [34, 39]. Since the arterial BP was suggested to be an important driving force pushing the MBF into tissues through arteriolar openings [17, 20], the whole-body distribution of the microcirculatory blood perfusion might be altered. Many treatment methods in Asian traditional medicine, such as Chinese herbs and acupuncture, have been noted to influence arterial wave transmission [40, 41].

The results of this study illustrate that the dilation of the underlying vessels and the decrease in the elastic modulus of the vessel walls during the application of AS might be greater at Hegu than at the nearby nonacupoints, possibly via a decrease in SNA as conjectured above. Consequently, not only is the local blood flow increased more at Hegu than at the nearby nonacupoints, but also the ability to change the arterial wave transmission and hence to adjust the whole-body blood distribution might also be greater in the MVB underlying Hegu than in the MVBs of the nearby nonacupoints. This may partly account for the physiological mechanism of the application of AS at the acupoints. Therefore, our results suggest that the application of AS to acupoints induces a more prominent effect in improving the microcirculation.

In conclusion, needling the Hegu acupoint significantly increased perfusion at Hegu but not at nearby nonacupoints. Spectral analysis of the blood-flow signal revealed that differences in SNA between Hegu and its nearby nonacupoints might account for this change in the microcirculation. To the best of our knowledge, this is the first time that this technique has been used to investigate the microcirculatory responses induced by AS. Our results suggest that the described noninvasive method can be used to compare sympathetic control of peripheral vascular activity between acupoints and nonacupoints, which might be important for studying the therapeutic effects of AS.

## Funding

National Science Council (partial).
